# Estimating the cure proportion of colorectal cancer and related factors after surgery in patients using parametric cure models 

**Published:** 2020

**Authors:** Neda Izadi, Fatemeh Koohi, Mahdi Safarpour, Parisa Naseri, Salar Rahimi, Soheila Khodakarim

**Affiliations:** 1 *Student Research Committee, Department of Epidemiology, School of Public Health and Safety, Shahid Beheshti University of Medical Sciences, Tehran, Iran*; 2 *Department of Epidemiology, School of Public Health and Safety, Shahid Beheshti University of Medical Sciences, Tehran, Iran *; 3 *Cellular and Molecular Research Center, Research Institute for Endocrine Sciences, Shahid Beheshti University of Medical Sciences, Tehran, Iran *; 4 *Department of Biostatistics, Faculty of Paramedical Sciences, Shahid Beheshti University of Medical Sciences, Tehran, Iran*; 5 *Colorectal Research Center, Shiraz University of Medical Sciences, Fars, Iran*; 6 *Department of Epidemiology, School of Public Health and Safety, School of Paramedical Science, Shahid Beheshti University of Medical Sciences, Tehran, Iran*

**Keywords:** Cure proportion, Related factors, Colorectal cancer, Parametric cure model

## Abstract

**Aim:**

This study aimed to estimate the cure proportion and effects of related factors on colorectal cancer in Iranian patients after surgery.

**Background:**

Colorectal cancer (CRC) is the third most commonly diagnosed cancer and the fourth leading cause of cancer death. The relative survival of CRC varies worldwide given the quality of care, including surgical techniques.

**Methods:**

This retrospective cohort study was conducted on 490 patients, aged 20–94 years, with colorectal cancer. All the colorectal cancer patients undergoing surgery in Faghihi hospital, Shiraz University of Medical Sciences were prospectively followed-up for 8 years from 2008 to March 8, 2016. We used parametric cure model (mixture and non-mixture) to estimate the cure proportion and the adjusted hazard ration (HR) for colorectal cancer mortality after surgery. Data were analyzed by the “flexsurvcure” package in R software (version 3.4.2).

**Results:**

The median age of patients was 57.5 (interquartile range =18) years. Specifically, 56.33% of the patients were male. The median time of follow-up in patients was 618 days. The cumulative survival proportion varied from 0.90 to 0.49 which indicated a reduction followed by a flat line in the probability of survival by sex. The flexible survival for adjusted cure proportion (%) was 68.3. Only obesity was associated with a decreased risk of mortality (HR=0.34; 95% CI: 0.12-0.97).

**Conclusion:**

The overall eight-year survival proportion and adjusted cure proportion for CRC were 49% and 68.3%, respectively. Knowing the cure proportion and its related factors in patients with CRC, better services can be provided. Thus, early detection and screening strategies are required to reduce mortality and increase survival of patients.

## Introduction

 Colorectal cancer (CRC) is the third most commonly diagnosed cancer in men and the second in women (1), and the fourth leading cause of cancer death worldwide (2). About two-thirds of colorectal cancer cases occur in countries characterized by high or very high indices of development (2). The global burden of CRC is predicted to rise by 60% to more than 2.2 million new cases and 1.1 million cancer deaths in 2030 (3). In Iran, according to the annual report of The National Cancer Registry Program in Iran (INCRS), CRC is the fourth common cancer in men and the second among women (4). Although the incidence of colorectal cancer in Iran is low compared to western countries, it seems to have witnessed a significant increase in the past decade (4).

The relative survival of CRC varies around the world even the quality of care, including surgical techniques (5). Relative survival analysis presents survival estimates at specific time points after diagnosis (6). A more accurate picture of the long-term outcome of cancer patients is estimating the proportion of cancer patients that are “statistically cured” (7). These measures are of interest to patients, physicians, and policymakers, and can offer insight into temporal trends in cancer patient survival (8). 

Age and tumor stage at diagnosis are known to strongly influence the ‘cured’ proportion of colorectal cancer and median survival time (7, 9). The studies of a cure for colorectal cancer patients have shown that the proportion of patients cured and median survival time of the uncured would diminish with advancing stage and increasing age (7, 10-12). This study aimed to estimate the cure proportion and effects of related factors on colorectal cancer in Iranian patients after surgery. 

## Methods

**Data Source and Data Collection**

This retrospective cohort study was conducted on 490 patients, aged 20–94 years, with colorectal cancer (ICD-10, C18–C20). All the colorectal cancer patients undergoing surgery in Faghihi hospital, Shiraz University of Medical Sciences were prospectively followed-up for 8 years from 2008 to March 8, 2016. Data were obtained from the Colorectal Research Center of Shiraz University of Medical Sciences, which registers information of all patients with colon and rectum malignancies. The information within patient's medical record in the Colorectal Surgery Department of Faghihi Hospital in Shiraz, including the history of the disease, the familial history, and data on paraclinical and diagnostic procedures as well as information on the type of surgery and its possible complications is registered in the Colorectal Research Center's electronic system. The information of each patient is reviewed and updated based on the time protocol of the Colorectal Research Center. Every patient was visited in the first year after surgery, every three months once, in the second year, every six months once and thereafter annually by the colorectal surgeon and their data were registered in the database of the Colorectal Research Center. In the absence of any patient referral, the patient’s death, or referring to another treatment center for follow-up treatment, the research center's staff contacted the patient or patient's family at certain intervals and they attempted to collect the necessary medical records and information of the patient's latest condition. 

In the current study, all patients who had been diagnosed with colorectal cancer, had undergone surgery, did not have any other cancers in other parts of the body and had complete information concerning the study factors at baseline were eligible for analysis. In addition, censored cases with less than 30 days of follow-up were excluded. In this study, death due to colorectal cancer was considered as the event with the time interval between the CRC’s surgery and CRC-related death calculated as the survival time of patients with CRC. In addition, the patients who survived after the lengthiest event time were identified as statistically cure patients.

The center's registry variables used to estimate cure in this study included age, sex, body mass index (BMI), size of tumor, location of tumor, grading, residual tumor after chemo-radiotherapy, staging, type of surgery, T-stage, N-stage, M-stage, obstruction, perforation, appearance, depth of invasion, vascular invasion, neural invasion, and lymphatic invasion.

In addition, according to the world health organization (WHO) guidelines, patients with BMI<18.5 kg/m2 were underweight, subjects with 18.5≤BMI<25 kg/m2 had normal weight, individuals with 25≤BMI<30 kg/m2 were overweight, and patients with BMI≤30 kg/m2 were obese (13). N-stage was also categorized into N1 (for N1(a,b,c) and N2(a,b)) and N0.

**Statistical Analysis**

Regarding the non-normal distribution of quantitative variables, median (Interquartile Range= IQR) and count (percentage) were used to describe quantitative and qualitative variables, respectively. The distributions of age, BMI, size of tumor, and number of lymph nodes among males and females were calculated by Mann-Whitney test, while the frequency of smoking and pathology reports among males and females was calculated by Chi-square test.

We implemented multiple imputations to impute values for variables with missing information using related covariates and used parametric cure model (mixture and non-mixture) to estimate the cure proportion and the adjusted hazard ratio (HR) for colorectal cancer mortality after surgery. 

In general, the parametric cure model includes two mixture and non-mixture cure fraction models. The mixture model for lifetime data sets assumes that the probability of the time-to-event is greater than a specified time t is given by the survival function: S(t)=p+(1-p)S0(t), where p is a parameter which represents the proportion of “long-term survivors” or “cured patients”, regarding the event of interest. The common choices for S0(t) are the Gompertz, exponential, and Weibull distributions.

In addition, the non-mixture model defines an asymptote for the cumulative hazard and hence for the cure fraction. In this case, the survival function is given by: S(t)=pF0(t)=exp[ln(p)F0(t)], where F0(t)=1−S0(t) (14). 

**Table 1:** Distribution of age, BMI, size of tumor, number of lymph nodes, and time of follow-up by sex among colorectal cancer patients (2008-2016)

Variable	Male (n=276)	Female (n=214)	Total (n=490)	P-value^*^
	Median (Q1-Q3)	Median (Q1-Q3)	Median (Q1-Q3)	
Age (year)	59 (50-70)	56 (46-62)	57.5 (47-65)	<0.001
BMI (kg/m^2^)	23.3 (20.4-26.5)	23.9 (21.1-27.3)	23.5 (20.8-26.7)	0.103
Size of tumor (cm)	3.5 (1.8-5)	3.5 (2-5)	3.5 (2-5)	0.862
Number of lymph nodes	7 (2-12)	8 (3-12)	7 (3-12)	0.184
Time of follow up (day)	618 (199.5-1255)	711.5 (233-1279)	670 (213-1259)	0.556

* Based on Mann-Whitney test, BMI=Body Mass Index

**Figure 1:**
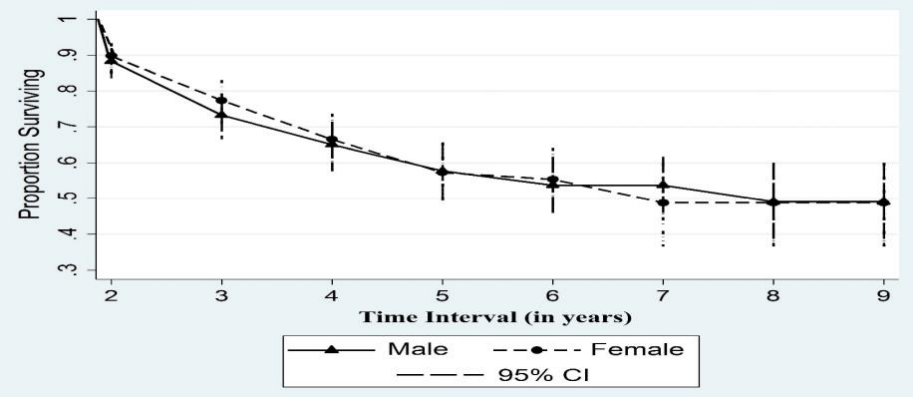
Cumulative survival proportion of colorectal cancer after surgery in patients by sex during 2008–2016

All the variables with a p-value of less than 0.3 were introduced into the regression model. In addition, we assumed the survival function follows a Weibull distribution for mixture and non-mixture models for both of which we used logit link function. The mixture and non-mixture models were fitted to the data and the best model was selected based on the AIC and BIC criteria. Data were analyzed by the Stata (version 12) and “flexsurvcure” function in “flexsurvcure” package (2017) in R software (version 3.4.2) (15). For all statistical tests, p<0.05 was considered statistically significant.

The present study was conducted according to the Helsinki Declaration. The study was approved by the ethics committee of the vice chancellery of research and technology, Shiraz University of Medical Science. Further, informed consent was obtained from patients or their families. 

## Results

The median age of 490 patients was 57.5 (IQR=18) years (range from 20 to 94 years). Specifically, 56.33% (276) of patients were male (sex ratio: 1.3 M/F). The type of disease in 60.41%, 38.16%, and 1.43% of patients was rectal, colon, and colorectal, respectively. Also, 11.43% and 5.31% of patients (56 and 26 patients) reported a family history of colon and rectal cancer, respectively. The median time of follow-up in patients was 670 (IQR=1046) days. 

**Table 2:** The frequency of pathology reports by sex among colorectal cancer patients (2008-2016)

Variable		Male	Female	Total	P-value^*^
		No (%)	No (%)	No (%)	
Location of tumor	Rectum	91 (41.18)	61 (34.66)	152 (38.29)	0.359
	Sigmoid	36 (16.29)	35 (19.89)	71 (17.88)	
	Recto-sigmoid	49 (22.17)	38 (21.59)	87 (21.91)	
	Cecum	11 (4.98)	8 (4.55)	19 (4.79)	
	Right colon	14 (6.33)	7 (3.98)	21 (5.29)	
	Other	20 (9.05)	27 (15.33)	47 (11.83)	
	Total	221 (100)	176 (100)	397 (100)	
Obstruction	No	183 (82.81)	154 (86.03)	337 (84.25)	0.371
	Yes	38 (17.19)	25 (13.97)	63 (15.75)	
	Total	221 (100)	179 (100)	400 (100)	
Perforation	No	200 (90.91)	164 (91.62)	364 (91.23)	0.802
	Yes	20 (9.09)	15 (8.38)	35 (8.77)	
	Total	220 (100)	179 (100)	399 (100)	
Appearance	Ulcerative	83 (39.9)	59 (34.3)	142 (37.37)	0.006
	Polypoid	30 (14.42)	30 (17.44)	60 (15.79)	
	Fungating	25 (12.02)	38 (22.09)	63 (16.58)	
	Diffuse infiltrative	24 (11.54)	15 (8.72)	39 (10.26)	
	Other	46 (22.11)	30 (17.44)	76 (20)	
	Total	208 (100)	172 (100)	380 (100)	
Differentiation (grading)	Well diff	141 (64.38)	119 (67.61)	260 (65.82)	0.330
	Moderately diff	55 (25.11)	40 (22.73)	96 (24.05)	
	Poorly diff	21 (9.59)	15 (8.52)	36 (9.11)	
	Other	2 (0.91)	2 (1.14)	4 (1.02)	
	Total	219 (100)	176 (100)	395 (100)	
Depth of invasion	Muscularis	78 (35.78)	59 (33.71)	137 (34.86)	0.616
	Serosal	129 (59.17)	100 (57.14)	229 (58.27)	
	Sub mucosal	9 (4.13)	13 (7.43)	22 (5.6)	
	Other	2 (0.92)	3 (1.71)	5 (1.27)	
	Total	218 (100)	184 (100)	393(100)	
Vascular invasion	No	206 (93.21)	166 (94.86)	372 (93.94)	0.490
	Yes	15 (6.79)	9 (5.14)	24 (6.06)	
	Total	221 (100)	175 (100)	396 (100)	
Neural invasion	No	190 (85.97)	151 (86.29)	341 (86.11)	0.925
	Yes	31 (14.03)	24 (13.71)	55 (13.89)	
	Total	221 (100)	175 (100)	396 (100)	
Lymphatic invasion	No	185 (83.71)	139 (79.43)	324 (81.82)	0.271
	Yes	36 (16.29)	36 (20.57)	72 (18.18)	
	Total	221 (100)	175 (100)	396 (100)	
T-stage	T0	21 (8.71)	16 (8.16)	37 (8.47)	0.712
	T1	11 (4.56)	15 (7.65)	26 (5.95)	
	T2	63 (26.14)	58 (29.59)	121 (27.69)	
	T3	133 (55.19)	96 (48.98)	229 (52.4)	
	T4	11 (4.56)	8 (4.08)	19 (4.35)	
	Tx/Tis	2 (0.82)	3 (1.53)	5 (1.15)	
	Total	241 (100)	196 (100)	437 (100)	
N-stage	N0	170 (70.54)	135 (68.88)	305 (69.79)	0.441
	N1(a,b,c)	45 (18.67)	45 (22.95)	90 (20.59)	
	N2(a,b)	25 (10.37)	14 (7.14)	39 (8.92)	
	Nx	1 (0.41)	2 (1.02)	3 (0.68)	
	Total	241 (100)	196 (100)	437 (100)	
M-stage	Mx	236 (97.93)	187 (95.41)	423 (96.8)	0.306
	M1(a,b)	5 (2.07)	9 (4.59)	14 (3.21)	
	Total	241 (100)	196 (100)	437 (100)	
Residual tumor after chemo-radiotherapy	No	222 (90.24)	172 (86.43)	394 (88.54)	0.203
	Yes	24 (9.76)	13.57	51 (11.46)	
	Total	246 (100)	199 (100)	445 (100)	
Staging	0	23 (9.58)	19 (9.79)	42 (9.68)	0.287
	I	65 (27.08)	63 (32.47)	128 (29.49)	
	II (A,B,C)	82 (34.16)	59 (30.41)	141 (32.48)	
	III (A,B,C)	70 (29.16)	53 (27.31)	123 (28.34)	
	Total	240 (100)	194 (100)	434 (100)	
Type of surgery	Laparotomy	98 (35.51)	89 (41.78)	187 (38.24)	0.215
	Laparoscopy	157 (56.88)	114 (53.52)	271 (55.42)	
	Conversion	21 (7.61)	10 (4.69)	31 (6.34)	
	Total	276 (100)	213 (100)	489 (100)	

* Based on Chi-square test

**Table 3:** Cure Proportion (%) of colorectal cancer after surgery by different models in patients during 2008–2016

Models	Cure Proportion			
	Mixture		Non-Mixture	
	%	95% CI	%	95% CI
Model I	48.9	40.6-57.2	48.2	39.2-57.3
Model II	51.2	39.4-63	51.7	42-61.4
Model III	71.4	30.9-95.1	70.1	31.1-92.8
Model IV	69.4	21.1-95	68.3	23.9-90.3

I= without any predictor variable, II= adjusted for age group and sex, III= adjusted for residual tumor after chemo-radiotherapy, staging, type of surgery, T-stage and N-stage, IV= adjusted for age group, sex, BMI, size of the tumor, residual tumor after chemo-radiotherapy, staging, type of surgery, T-stage and N-stage

**Table 4:** Simple and multiple non-mixture model analysis of survival of patients with colorectal cancer (2008–2016)

Variable		Simple		Multiple	
		HR	95% CI	HR	95% CI
Age	<60 y	1	-	1	-
	≥ 60 y	1.04	0.74-1.45	0.96	0.65-1.41
Sex	Female	1	-	1	-
	Male	1.05	0.75-1.47	1.07	0.72-1.58
BMI	Obese	0.53	0.24-1.16	0.34	0.12-0.97
	Overweight	0.90	0.61-1.33	0.86	0.56-1.33
	Normal	1	-	1	-
	Underweight	1.09	0.65-1.84	1.06	0.61-1.84
Residual tumor after chemo-radio therapy	No	1	-	1	-
	Yes	1.08	0.59-1.96	1.73	0.75-3.98
Staging	0	1	-	1	-
	I	1.14	0.58-2.30	1.86	0.72-4.79
	II (A,B,C)	1.12	0.55-2.27	1.59	0.63-3.98
	III (A,B,C)	1.53	0.79-3.07	1.8	0.44-7.53
Type of surgery	Conversion	1	-	1	-
	Laparoscopy	0.81	0.39-1.69	0.74	0.33-1.66
	Laparotomy	1.48	0.71-3.10	1.47	0.65-3.31
N-stage	N0	1	-	1	-
	N1	1.41	0.98-2.03	1.32	0.45-3. 9
T-stage	T0	1	-	-	-
	T1	1.20	0.63-2.31	-	-
Size of tumor	< 3.5 cm	1	-	-	-
	≥ 3.5 cm	1.10	0.77-1.58	-	-
Vascular invasion	No	1	-	-	-
	Yes	1.34	0.59-3.06	-	-
Neural invasion	No	1	-	-	-
	Yes	1.67	0.77-3.49	-	-
Lymphatic invasion	No	1	-	-	-
	Yes	1.46	0.62-2.75	-	-

HR= Hazard Ratio; CI= Confidence Interval; P-value for overall multiple non-mixture model=0.046

In investigating the relationship between variables such as age, BMI,ize of the tumor, number of lymph nodes, as well as time of follow-up and sex, the only age was significantly higher in men than in women (Table 1). In addition, the qualitative variable (pathology reports) except appearance, did not show any significant difference with sexes (Table 2).

**Cumulative survival**

The cumulative survival proportion varied from 0.90 to 0.49, and indicated a decline followed by a flat line in the probability of survival by sex (Fig. 1). 

Cure proportion and risk of mortality after surgery:

The flexible survival cure was used for cure proportion, with the adjusted cure proportion (%) being 48.9 and 69.4 in the mixture and 48.2 and 68.3 in non-mixture cure models, respectively (Table 3). In investigating the variables associated with risk of mortality after surgery, including age, sex, BMI, size of tumor, residual tumor, staging, type of surgery, T-stage and N-stage, using unadjusted and adjusted non-mixture regression (based on lower AIC and BIC criteria for non-mixture model), hazard ratio did not show any significant relationship between the risk of mortality after surgery and different variables (P>0.05). Only obesity was associated with a decreased risk of mortality (OR=0.35; 95% CI: 0.12-0.98) (Table 4).

## Discussion

The overall eight-year survival proportion and adjusted cure proportion for CRC were 49% and 68.3%, respectively. A primary focus of most cancer research is to change the probability of cure and enhance the expected survival time for cured patients. It often happens that a fraction of subjects will never experience the event of interest and some patients will never suffer a relapse of a given disease (16, 17). These subjects are usually considered as having disease-free survival and are said to be cured. Thus, classical survival models have been extended to what is commonly referred to as cure models (16).

Cure models can determine risk factors with a significant effect on the survival of cured and uncured patients. These models would be beneficial as they can distinguish between clinical determinants of cure and variables associated with the survival (18). Accordingly, the parametric cure model was applied to colorectal cancer data collected as individual records including 490 cases followed during the period 2008-2016. 

The results revealed that sex was not significantly associated either with the proportion of cured patients or with excess risk in both simple and multiple non-mixture models. However, it has previously been shown that females have a better survival than males, which is mainly attributed to a higher cure proportion among females (19).

The same results were observed when age was added to the model as a covariate. Nevertheless, in multiple non-mixture models, the proportion of cured patients slightly increased in patients aged older than 60 years compared to younger patients. This change looked fairly different from the one observed in the simple model, but the result was not statistically different. The results reported in previous studies indicated that the probability of cure must logically diminish with increasing age since younger patients usually respond better to therapies than older patients do (20, 21). Reaching different results in this regard might be because of using Weibull function instead of other possible functions such as the exponential form to model the proportion of cured patients (21). 

The multiple non-mixture models estimated with the stage of cancer indicated a non-significant association between this covariate and the probability of cure after surgery. This result suggested that some un-staged patients in the reference group might have a non-localized tumor at the time of the diagnosis. Applying different diagnostic protocols using different definition of the stage might be one of the reasons for such a misclassification. This result emphasizes the importance of more detailed diagnostic and staging procedures to classify patients that would have been considered as un-staged in earlier times.

Note that the application and interpretation of the results derived from parametric cure models highly depend on assumptions, and violation of these assumptions may lead to incorrect interpretation. The key assumption is that the distribution of survival times in uncured cases must be described by parametric distribution functions (19). In the present study, the non-mixture model and the Weibull distribution presented a significantly better performance and provided a good fit to data compared to other models. Thus, the non-mixture model was assumed to suit stable estimates of the model parameters. 

The next point is that the ten-year follow-up can be considered to be beyond the minimum threshold required to level off the survival curves (22). Besides satisfying the assumptions and prerequisites for parametric cure models, no significant parameters could be determined either in simple or multiple models. 

One of the reasons for obtaining such results might be because of not including some risk factors contributing to the risk of colorectal cancer into the model. For example, diet and alcohol intake are the most important examples of risk factors associated with colorectal cancer as well as to other cancers such as the stomach, the rectum, and breast (23-25). Thus, it is believed that the probability of cure can drop in patients with high consumption of red meat and alcohol compared to those with a healthy regime (26). The lack of such clinically important variables can be mentioned as a possible limitation of the current study. Nevertheless, fairly large sample size and long-term follow-up can be considered as the strengths of this study.

The overall eight-year survival proportion for CRC was 49%. Although the cure proportion of colorectal cancer was the same for mixture and non-mixture in the crude model, the non-mixture model gave a better performance and hazard function and provided a good fit to data in multiple models. In addition, by knowing the cure proportion of CRC and its related factors in patients with CRC, better services can be provided. Thus, early detection and screening strategies are required to reduce mortality and increase survival of patients.
